# 3-Chloro-*N*′-(3,5-dibromo-2-hy­droxy­benzyl­idene)benzohydrazide methanol monosolvate

**DOI:** 10.1107/S1600536811000742

**Published:** 2011-01-12

**Authors:** Tian-Yi Li, Yue-Gang Qu

**Affiliations:** a School of Chemical Engineering, Changchun University of Technology, Changchun 130012, People’s Republic of China

## Abstract

The title Schiff base compound, C_14_H_9_Br_2_ClN_2_O_2_·CH_3_OH, features an intra­molecular O—H⋯N hydrogen bond, which contributes to the planarity of the mol­ecule: the dihedral angle between the two benzene rings is 4.6 (2)°. In the crystal, pairs of adjacent mol­ecules are linked through inter­molecular N—H⋯O and O—H⋯O hydrogen bonds, forming dimers. The methanol solvent mol­ecule is linked by inter­molecular O—H⋯O hydrogen bonds.

## Related literature

For Schiff base compounds derived from the reaction of aldehydes with benzohydrazides, see: Pouralimardan *et al.* (2007) ▶; Dinda *et al.* (2002 ▶); Podyachev *et al.* (2007 ▶). For reference bond lengths, see: Allen *et al.* (1987 ▶).
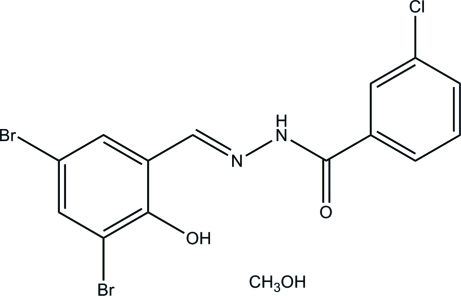


## Experimental

### 

#### Crystal data

C_14_H_9_Br_2_ClN_2_O_2_·CH_4_O
*M* *_r_* = 464.54
Triclinic, 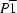

*a* = 8.8560 (18) Å
*b* = 9.3810 (19) Å
*c* = 11.205 (2) Å
α = 95.634 (3)°
β = 110.952 (3)°
γ = 99.392 (3)°
*V* = 845.2 (3) Å^3^
*Z* = 2
Mo *K*α radiation
μ = 4.97 mm^−1^
*T* = 298 K
0.18 × 0.17 × 0.17 mm

#### Data collection

Bruker APEXII CCD area-detector diffractometer
Absorption correction: multi-scan (*SADABS*; Bruker, 2005 ▶) *T*_min_ = 0.468, *T*_max_ = 0.486
7213 measured reflections
3504 independent reflections
2423 reflections with *I* > 2σ(*I*)
*R*_int_ = 0.036

#### Refinement

*R*[*F* ^2^ > 2σ(*F* ^2^)] = 0.044
*wR*(*F* ^2^) = 0.117
*S* = 1.03
3504 reflections
214 parameters
1 restraint
H atoms treated by a mixture of independent and constrained refinement
Δρ_max_ = 0.61 e Å^−3^
Δρ_min_ = −0.71 e Å^−3^

### 

Data collection: *APEX2* (Bruker, 2005 ▶); cell refinement: *SAINT* (Bruker, 2005 ▶); data reduction: *SAINT*; program(s) used to solve structure: *SHELXTL* (Sheldrick, 2008 ▶); program(s) used to refine structure: *SHELXTL* molecular graphics: *SHELXTL*; software used to prepare material for publication: *SHELXTL*.

## Supplementary Material

Crystal structure: contains datablocks global, I. DOI: 10.1107/S1600536811000742/hg2785sup1.cif

Structure factors: contains datablocks I. DOI: 10.1107/S1600536811000742/hg2785Isup2.hkl

Additional supplementary materials:  crystallographic information; 3D view; checkCIF report

## Figures and Tables

**Table 1:** Hydrogen-bond geometry (Å, °)

*D*—H⋯*A*	*D*—H	H⋯*A*	*D*⋯*A*	*D*—H⋯*A*
N2—H2⋯O3^i^	0.90 (4)	1.98 (2)	2.852 (4)	165 (5)
O3—H3⋯O2^ii^	0.82	1.98	2.769 (4)	161
O1—H1⋯N1	0.82	1.85	2.566 (4)	146

Symmetry codes: (i) 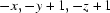; (ii) 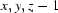.

**Fig. 1.**
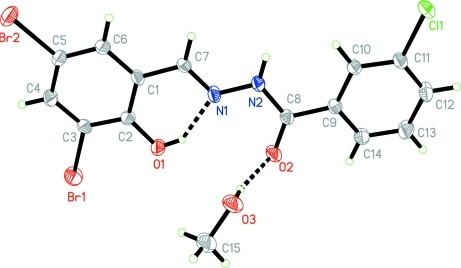
The molecular structure of the title compound with 30% probability displacement ellipsoids for non-hydrogen atoms. Hydrogen bonds are drawn as dashed lines.

**Fig. 2.**
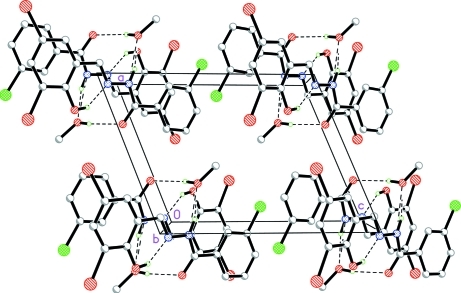
The molecular packing of the title compound. Hydrogen bonds are drawn as dashed lines.

## supplementary crystallographic information

### Comment

In the last few years, a number of Schiff bases derived from the reaction of aldehydes with benzohydrazides were prepared and structurally characterized (Pouralimardan *et al.*, 2007; Dinda *et al.*, 2002). As a continuation of the work, in the present paper, the structure of the title Schiff base compound, (Fig. 1) is reported.

In the title compound, there is an O—H···N hydrogen bond, which contributes to the planarity of the molecule. The dihedral angle between the two benzene rings is 4.6 (2)°. All the bond lengths are within normal values (Allen *et al.*, 1987). The adjacent two molecules are linked through intermolecular N—H···O and O—H···O hydrogen bonds (Table 1) to form a dimer (Fig. 2).   The methanol solvate is linked by intermolecular O—H···O hydrogen bonds.

### Experimental

3,5-dibromo-2-hydroxybenzaldehyde (0.280 g, 1 mmol) and 3-chlorobenzohydrazide (0.171 g, 1 mmol) were dissolved in 30 ml absolute methanol. The mixture was stirred at reflux for 10 min, and cooled to room temperature. The clear colorless solution was left to slow evaporation in air for three days, yielding colorless block-shaped crystals, which were collected by filtration and washed with methanol.

### Refinement

The amino H atom was located from a difference Fourier map and refined isotropically, with the N—H distance restrained to 0.90 (1) Å. The other H atoms were positioned geometrically and refined using the riding-model approximation, with C—H = 0.93 or 0.96 Å, and O—H = 0.82 Å, and *U*_iso_(H) = 1.2*U*_eq_(C) or *U*_iso_(H) = 1.5*U*_eq_(C15 and O).

### Crystal data

**Table:**

C_14_H_9_Br_2_ClN_2_O_2_·CH_4_O	*Z* = 2
*M**_r_* = 464.54	*F*(000) = 456
Triclinic, *P*1	*D*_x_ = 1.825 Mg m^−^^3^
Hall symbol: -P 1	Mo *K*α radiation, λ = 0.71073 Å
*a* = 8.8560 (18) Å	Cell parameters from 2168 reflections
*b* = 9.3810 (19) Å	θ = 2.5–25.9°
*c* = 11.205 (2) Å	µ = 4.97 mm^−^^1^
α = 95.634 (3)°	*T* = 298 K
β = 110.952 (3)°	Block, colorless
γ = 99.392 (3)°	0.18 × 0.17 × 0.17 mm
*V* = 845.2 (3) Å^3^	

### Data collection

**Table:**

Bruker APEXII CCD area-detector diffractometer	3504 independent reflections
Radiation source: fine-focus sealed tube	2423 reflections with *I* > 2σ(*I*)
graphite	*R*_int_ = 0.036
ω scans	θ_max_ = 27.0°, θ_min_ = 2.0°
Absorption correction: multi-scan (*SADABS*; Bruker, 2005)	*h* = −11→11
*T*_min_ = 0.468, *T*_max_ = 0.486	*k* = −11→11
7213 measured reflections	*l* = −14→13

### Refinement

**Table:**

Refinement on *F*^2^	Primary atom site location: structure-invariant direct methods
Least-squares matrix: full	Secondary atom site location: difference Fourier map
*R*[*F*^2^ > 2σ(*F*^2^)] = 0.044	Hydrogen site location: inferred from neighbouring sites
*wR*(*F*^2^) = 0.117	H atoms treated by a mixture of independent and constrained refinement
*S* = 1.03	*w* = 1/[σ^2^(*F*_o_^2^) + (0.050*P*)^2^ + 0.0441*P*] where *P* = (*F*_o_^2^ + 2*F*_c_^2^)/3
3504 reflections	(Δ/σ)_max_ < 0.001
214 parameters	Δρ_max_ = 0.61 e Å^−^^3^
1 restraint	Δρ_min_ = −0.71 e Å^−^^3^

### Special details

**Table:**

Geometry. All e.s.d.'s (except the e.s.d. in the dihedral angle between two l.s. planes) are estimated using the full covariance matrix. The cell e.s.d.'s are taken into account individually in the estimation of e.s.d.'s in distances, angles and torsion angles; correlations between e.s.d.'s in cell parameters are only used when they are defined by crystal symmetry. An approximate (isotropic) treatment of cell e.s.d.'s is used for estimating e.s.d.'s involving l.s. planes.
Refinement. Refinement of *F*^2^ against ALL reflections. The weighted *R*-factor *wR* and goodness of fit *S* are based on *F*^2^, conventional *R*-factors *R* are based on *F*, with *F* set to zero for negative *F*^2^. The threshold expression of *F*^2^ > σ(*F*^2^) is used only for calculating *R*-factors(gt) *etc*. and is not relevant to the choice of reflections for refinement. *R*-factors based on *F*^2^ are statistically about twice as large as those based on *F*, and *R*- factors based on ALL data will be even larger.

### Fractional atomic coordinates and isotropic or equivalent isotropic displacement parameters (Å^2^)

**Table:**

	*x*	*y*	*z*	*U*_iso_*/*U*_eq_	
Br1	0.29523 (6)	0.00200 (5)	1.38779 (5)	0.0684 (2)	
Br2	−0.39950 (6)	−0.16848 (6)	1.18451 (5)	0.0754 (2)	
Cl1	−0.12376 (13)	0.68386 (11)	0.50390 (9)	0.0538 (3)	
N1	0.0467 (4)	0.2942 (3)	0.9916 (3)	0.0375 (7)	
N2	0.0405 (4)	0.3784 (3)	0.8978 (3)	0.0386 (7)	
O1	0.2266 (3)	0.1922 (3)	1.1849 (3)	0.0496 (7)	
H1	0.2078	0.2450	1.1295	0.074*	
O2	0.3090 (3)	0.4803 (3)	1.0115 (3)	0.0533 (7)	
O3	0.2945 (3)	0.6052 (3)	0.2405 (3)	0.0578 (8)	
H3	0.3060	0.5530	0.1826	0.087*	
C1	−0.0709 (5)	0.1171 (4)	1.0859 (3)	0.0338 (8)	
C2	0.0834 (4)	0.1111 (4)	1.1785 (3)	0.0346 (8)	
C3	0.0872 (4)	0.0173 (4)	1.2683 (3)	0.0388 (8)	
C4	−0.0538 (5)	−0.0650 (4)	1.2702 (3)	0.0439 (9)	
H4	−0.0484	−0.1259	1.3316	0.053*	
C5	−0.2040 (5)	−0.0563 (4)	1.1797 (4)	0.0420 (9)	
C6	−0.2136 (5)	0.0304 (4)	1.0871 (3)	0.0392 (8)	
H6	−0.3162	0.0315	1.0246	0.047*	
C7	−0.0839 (4)	0.2091 (4)	0.9867 (3)	0.0356 (8)	
H7	−0.1856	0.2058	0.9212	0.043*	
C8	0.1846 (4)	0.4709 (4)	0.9153 (3)	0.0349 (8)	
C9	0.1854 (4)	0.5574 (4)	0.8114 (3)	0.0341 (8)	
C10	0.0427 (4)	0.5745 (3)	0.7138 (3)	0.0349 (8)	
H10	−0.0608	0.5274	0.7088	0.042*	
C11	0.0560 (5)	0.6615 (4)	0.6248 (3)	0.0388 (8)	
C12	0.2061 (5)	0.7299 (4)	0.6284 (4)	0.0503 (10)	
H12	0.2126	0.7875	0.5667	0.060*	
C13	0.3475 (5)	0.7129 (5)	0.7242 (4)	0.0560 (11)	
H13	0.4504	0.7598	0.7279	0.067*	
C14	0.3379 (5)	0.6269 (4)	0.8146 (4)	0.0459 (9)	
H14	0.4345	0.6151	0.8785	0.055*	
C15	0.4103 (6)	0.5924 (6)	0.3610 (4)	0.0718 (14)	
H15A	0.5198	0.6338	0.3671	0.108*	
H15B	0.4020	0.4907	0.3691	0.108*	
H15C	0.3882	0.6438	0.4293	0.108*	
H2	−0.060 (3)	0.383 (5)	0.842 (4)	0.086*	

### Atomic displacement parameters (Å^2^)

**Table:**

	*U*^11^	*U*^22^	*U*^33^	*U*^12^	*U*^13^	*U*^23^
Br1	0.0442 (3)	0.0852 (4)	0.0707 (3)	0.0200 (2)	0.0048 (2)	0.0458 (3)
Br2	0.0454 (3)	0.0852 (4)	0.0891 (4)	−0.0070 (2)	0.0186 (3)	0.0470 (3)
Cl1	0.0495 (6)	0.0638 (6)	0.0440 (6)	0.0160 (5)	0.0074 (5)	0.0251 (5)
N1	0.0416 (18)	0.0382 (16)	0.0383 (17)	0.0134 (14)	0.0164 (15)	0.0195 (13)
N2	0.0362 (18)	0.0394 (16)	0.0402 (18)	0.0099 (14)	0.0104 (15)	0.0198 (14)
O1	0.0347 (15)	0.0582 (17)	0.0538 (17)	0.0050 (13)	0.0113 (13)	0.0294 (13)
O2	0.0344 (15)	0.078 (2)	0.0455 (16)	0.0117 (14)	0.0079 (13)	0.0314 (14)
O3	0.0395 (16)	0.079 (2)	0.0466 (17)	0.0142 (15)	0.0039 (14)	0.0187 (15)
C1	0.042 (2)	0.0317 (18)	0.0286 (18)	0.0103 (15)	0.0115 (16)	0.0120 (14)
C2	0.0297 (19)	0.0355 (19)	0.039 (2)	0.0093 (15)	0.0112 (17)	0.0104 (15)
C3	0.036 (2)	0.041 (2)	0.0352 (19)	0.0115 (17)	0.0050 (17)	0.0148 (16)
C4	0.046 (2)	0.041 (2)	0.046 (2)	0.0125 (18)	0.015 (2)	0.0217 (18)
C5	0.037 (2)	0.041 (2)	0.046 (2)	0.0011 (17)	0.0141 (19)	0.0136 (17)
C6	0.033 (2)	0.042 (2)	0.040 (2)	0.0082 (17)	0.0081 (17)	0.0134 (16)
C7	0.033 (2)	0.0370 (19)	0.0334 (19)	0.0102 (16)	0.0061 (16)	0.0127 (15)
C8	0.0312 (19)	0.0416 (19)	0.036 (2)	0.0130 (16)	0.0136 (17)	0.0146 (15)
C9	0.036 (2)	0.0349 (19)	0.0340 (19)	0.0109 (16)	0.0136 (17)	0.0081 (15)
C10	0.032 (2)	0.039 (2)	0.0345 (19)	0.0081 (16)	0.0123 (17)	0.0110 (16)
C11	0.043 (2)	0.038 (2)	0.036 (2)	0.0135 (17)	0.0119 (18)	0.0121 (16)
C12	0.054 (3)	0.054 (2)	0.056 (2)	0.018 (2)	0.028 (2)	0.029 (2)
C13	0.037 (2)	0.068 (3)	0.070 (3)	0.008 (2)	0.026 (2)	0.031 (2)
C14	0.031 (2)	0.057 (2)	0.052 (2)	0.0115 (18)	0.0152 (19)	0.0227 (19)
C15	0.045 (3)	0.104 (4)	0.055 (3)	0.007 (3)	0.005 (2)	0.031 (3)

### Geometric parameters (Å, °)

**Table:**

Br1—C3	1.889 (3)	C4—H4	0.9300
Br2—C5	1.895 (4)	C5—C6	1.368 (5)
Cl1—C11	1.743 (4)	C6—H6	0.9300
N1—C7	1.274 (4)	C7—H7	0.9300
N1—N2	1.367 (4)	C8—C9	1.484 (4)
N2—C8	1.359 (5)	C9—C14	1.387 (5)
N2—H2	0.90 (4)	C9—C10	1.389 (4)
O1—C2	1.342 (4)	C10—C11	1.374 (4)
O1—H1	0.8200	C10—H10	0.9300
O2—C8	1.218 (4)	C11—C12	1.363 (6)
O3—C15	1.404 (5)	C12—C13	1.373 (6)
O3—H3	0.8200	C12—H12	0.9300
C1—C6	1.392 (5)	C13—C14	1.373 (5)
C1—C2	1.403 (5)	C13—H13	0.9300
C1—C7	1.457 (4)	C14—H14	0.9300
C2—C3	1.396 (5)	C15—H15A	0.9600
C3—C4	1.366 (5)	C15—H15B	0.9600
C4—C5	1.375 (5)	C15—H15C	0.9600
			
C7—N1—N2	120.4 (3)	O2—C8—N2	121.2 (3)
C8—N2—N1	115.9 (3)	O2—C8—C9	121.3 (3)
C8—N2—H2	125 (3)	N2—C8—C9	117.4 (3)
N1—N2—H2	118 (3)	C14—C9—C10	118.8 (3)
C2—O1—H1	109.5	C14—C9—C8	117.5 (3)
C15—O3—H3	109.5	C10—C9—C8	123.7 (3)
C6—C1—C2	119.2 (3)	C11—C10—C9	119.4 (3)
C6—C1—C7	119.5 (3)	C11—C10—H10	120.3
C2—C1—C7	121.3 (3)	C9—C10—H10	120.3
O1—C2—C3	118.8 (3)	C12—C11—C10	121.7 (3)
O1—C2—C1	123.0 (3)	C12—C11—Cl1	119.3 (3)
C3—C2—C1	118.3 (3)	C10—C11—Cl1	119.0 (3)
C4—C3—C2	122.0 (3)	C11—C12—C13	119.2 (3)
C4—C3—Br1	119.6 (3)	C11—C12—H12	120.4
C2—C3—Br1	118.4 (3)	C13—C12—H12	120.4
C3—C4—C5	118.8 (3)	C12—C13—C14	120.4 (4)
C3—C4—H4	120.6	C12—C13—H13	119.8
C5—C4—H4	120.6	C14—C13—H13	119.8
C6—C5—C4	121.2 (3)	C13—C14—C9	120.5 (4)
C6—C5—Br2	120.2 (3)	C13—C14—H14	119.7
C4—C5—Br2	118.6 (3)	C9—C14—H14	119.7
C5—C6—C1	120.4 (4)	O3—C15—H15A	109.5
C5—C6—H6	119.8	O3—C15—H15B	109.5
C1—C6—H6	119.8	H15A—C15—H15B	109.5
N1—C7—C1	118.7 (3)	O3—C15—H15C	109.5
N1—C7—H7	120.6	H15A—C15—H15C	109.5
C1—C7—H7	120.6	H15B—C15—H15C	109.5

### Hydrogen-bond geometry (Å, °)

**Table:**

*D*—H···*A*	*D*—H	H···*A*	*D*···*A*	*D*—H···*A*
N2—H2···O3^i^	0.90 (4)	1.98 (2)	2.852 (4)	165 (5)
O3—H3···O2^ii^	0.82	1.98	2.769 (4)	161
O1—H1···N1	0.82	1.85	2.566 (4)	146

Symmetry codes: (i) −*x*, −*y*+1, −*z*+1; (ii) *x*, *y*, *z*−1.
